# A *HUWE1* defect causes PARP inhibitor resistance by modulating the BRCA1-∆11q splice variant

**DOI:** 10.1038/s41388-023-02782-8

**Published:** 2023-07-25

**Authors:** Stephen J. Pettitt, Nan Shao, Diana Zatreanu, Jessica Frankum, Ilirjana Bajrami, Rachel Brough, Dragomir B. Krastev, Theodoros I. Roumeliotis, Jyoti S. Choudhary, Sonja Lorenz, Alistair Rust, Johann S. de Bono, Timothy A. Yap, Andrew N. J. Tutt, Christopher J. Lord

**Affiliations:** 1 The CRUK Gene Function Laboratory, The Breast Cancer Now Toby Robins Research Centre, The Institute of Cancer Research, London, SW3 6JB UK; 2 The Institute of Cancer Research, London, SW3 6JB UK; 3 Max Planck Institute for Multidisciplinary Sciences, 37077 Göttingen, Germany; 4 The Institute of Cancer Research, The Royal Marsden Hospital, Downs Road, Sutton, Surrey, SM2 5PT UK; 5 Present Address: University of Texas MD Anderson Cancer Center, 1400 Holcombe Blvd, Houston, TX 77030 USA

**Keywords:** Cancer genomics, Ovarian cancer, Homologous recombination

## Abstract

Although PARP inhibitors (PARPi) now form part of the standard-of-care for the treatment of homologous recombination defective cancers, de novo and acquired resistance limits their overall effectiveness. Previously, overexpression of the BRCA1-∆11q splice variant has been shown to cause PARPi resistance. How cancer cells achieve increased BRCA1-∆11q expression has remained unclear. Using isogenic cells with different *BRCA1* mutations, we show that reduction in HUWE1 leads to increased levels of BRCA1-∆11q and PARPi resistance. This effect is specific to cells able to express BRCA1-∆11q (e.g. *BRCA1* exon 11 mutant cells) and is not seen in *BRCA1* mutants that cannot express BRCA1-∆11q, nor in *BRCA2* mutant cells. As well as increasing levels of BRCA1-∆11q protein in exon 11 mutant cells, HUWE1 silencing also restores RAD51 nuclear foci and platinum salt resistance. HUWE1 catalytic domain mutations were also seen in a case of PARPi resistant, *BRCA1* exon 11 mutant, high grade serous ovarian cancer. These results suggest how elevated levels of BRCA1-∆11q and PARPi resistance can be achieved, identify HUWE1 as a candidate biomarker of PARPi resistance for assessment in future clinical trials and illustrate how some PARPi resistance mechanisms may only operate in patients with particular *BRCA1* mutations.

## Introduction

PARP inhibitors (PARPi) are now approved for the treatment of breast, ovarian, pancreatic or prostate cancers with defects in the homologous recombination (HR) DNA repair pathway [1]. For example, the efficacy of PARP inhibitors in tumours with germline *BRCA1* or *BRCA2* mutations has been well established, in both metastatic and early disease settings in breast and ovarian cancer [2–8]. However, many tumours display either pre-existing or acquired resistance to PARPi, limiting their efficacy and pointing to a need to further understand how clinical PARPi resistance emerges [9].

The most well established mechanism of clinical PARP inhibitor resistance is reversion mutation, whereby a second mutation restores the HR function of *BRCA1* or *BRCA2* [10–13]. This may occur in up to 40% of patients [9], although estimates vary based on clinical setting. Other potential resistance mechanisms have been identified in the laboratory, including *PARP1* or *PARG* loss [14–16], defects in the double strand break protecting 53BP1-Shieldin [17–22] or CST complexes [23, 24] and upregulation of drug transporter pumps [25, 26]. However, thus far there is limited clinical evidence of mutations in these pathways in resistant tumours. This may be due to a lack of sequencing data covering these genes in resistant biopsies, non-mutational disruption of these pathways or differences between patients and preclinical model systems. Nevertheless, a large fraction of PARPi resistance remains to be explained.

One additional mechanism that has been described in preclinical experiments is stabilisation or increased expression of splice variants of *BRCA1/2* that exclude the pathogenic mutation but retain some function. In addition, some reversion mutations affect splice sites adjacent to the exon encoding the pathogenic mutation, implying that alterations of splicing to bypass the pathogenic mutation could be occurring [10, 27–30]. For example, the RING domain mutation in the N-terminus of *BRCA1* can be bypassed by expression of a hypomorphic “RINGless” BRCA1 protein [31–33]. MDA-MB-436 cells selected for resistance to PARP inhibitors also display HSP90-dependent expression of a hypomorphic protein [34]. Expression of the *BRCA1* splice variant ∆11q, which excludes most of exon 11 has also been shown to be sufficient for PARP inhibitor resistance in *BRCA1* knockout or mutant cell lines [35, 36]. Although referred to as exon 11 for historical reasons, this is actually exon 10 in the canonical transcript of *BRCA1* and is the largest exon, comprising 60% of the coding sequence. This sequence is not required for cellular PARPi resistance: cells with exon 11 mutations show further increases in PARP inhibitor sensitivity when BRCA1 is silenced by RNA interference [14] or when mutations are made in other, constitutively spliced, exons of BRCA1 [35]. Large deletions in exon 11 have also been observed in clinical *BRCA1* reversion mutations [10]. How the expression of BRCA1-∆11q is regulated in *trans*, and whether this occurs in patients, has not been established.

## Results

### HUWE1 is required for turnover of the BRCA1-∆11q protein

**Fig. 1:**
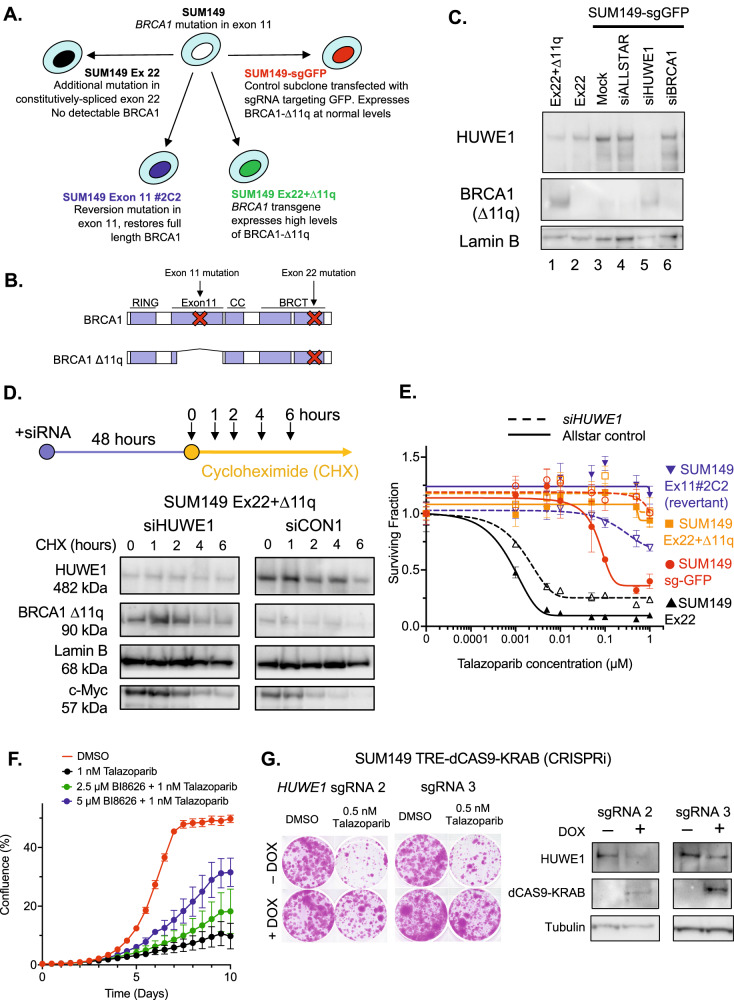
*HUWE1* is required for BRCA1*-*∆11q stability and PARPi sensitivity. **A** SUM149 isogenic series. *BRCA1* mutations and/or reversions in each cell line are described. **B** Diagram of wild type or ∆11q BRCA1 protein structures. Location of mutations in SUM149 cell line series in context of protein domains are shown. **C** Cancer cell lines harboring mutations in the BRCA1 exon11 express a BRCA1-△ex11 splice variant, ∆11q, lacking the majority of exon 11. Western blot showing expression of BRCA1 proteins in nuclear extracts, detected using a N terminal antibody. Effect of HUWE1 gene silencing on ∆11q levels is also shown. **D** HUWE1 silencing stabilizes the BRCA1 ∆11q protein. BRCA1 Ex22 + ∆11q cells were transfected with HUWE1 or non-targeting siRNA as indicated. After 48 h, cells were exposed to 150 µg/ml cycloheximide for 0–6 h as indicated. Stability was determined for the indicated proteins using Western blot. Lamin B is included as a loading control; c-Myc is a canonical HUWE1 substrate. Whole cell lysates were used in all the samples. **E** HUWE1 gene silencing promotes partial PARPi resistance in SUM149 sg-GFP cells. Indicated cells were transfected with siRNA as shown and then 48 h later, exposed to PARP inhibitors for a subsequent five days. Cell survival was measured using CellTiter Glo. For SUM149 sg-GFP cells, siHUWE1 *vs*. Allstar (non-targeting) control, ANOVA *p* < 0.0001. **F** A toolbox HUWE1 inhibitor, BI8626, reduces PARPi sensitivity in BRCA1 exon 11 mutant SUM149 cells. Cells were exposed to the indicated drugs and confluency measured using Incucyte live cell imaging. Error bars represent standard deviation from triplicate experiments. **G** Silencing of HUWE1 expression using CRISPR interference (CRISPRi) leads to PARPi resistance. SUM149 TRE-dCas9-KRAB cells were transfected with the indicated sgRNA vectors and dCas9-KRAB expression induced by exposure to doxycycline. Cells were exposed to talazoparib or equivalent DMSO solvent as indicated, and clonogenic survival estimated by crystal violet staining after 14 days. Western blots showing induction of dCas9-KRAB and reduction in HUWE1 levels after 48 h exposure to doxycycline are shown to the right.

Since full-length BRCA1 protein is a known substrate of the ubiquitin ligase HUWE1 [37–39], we considered whether a reduction in HUWE1 activity might also stabilise BRCA1 splice variants such as BRCA1-∆11q. To test this hypothesis, we used a previously-described panel of cell lines derived from BRCA1 mutant SUM149 triple negative breast cancer tumour cells [35] (Fig. 1A, B); SUM149 has a pathogenic exon 11 frameshift mutation (*BRCA1*:c.2169delT). The SUM149 derivates were: (i) a derivative with an additional CRISPR-generated BRCA1 mutation in the constitutively-spliced exon 22, termed “SUM149 Ex22”. Because of the exon 22 truncating mutation, SUM149 Ex22 is also unable to express the BRCA1-∆11q protein; (ii) a SUM149 derivative with a reversion mutation in exon 11 which restores full length *BRCA1* expression (“SUM149 Ex11#2C2”); (iii) a SUM149 derivative with a BRCA1 transgene that ectopically expresses high levels of BRCA1-∆11q (SUM149 Ex22 + ∆11q [35]); or (iv) a control subclone transfected with sgRNA targeting GFP (SUM149-sgGFP) that is able to expresses BRCA1-∆11q and does so at relatively low levels [35]. These cell lines show a spectrum of PARPi sensitivity; the revertant SUM149 Ex11#2C2 and Ex22 + ∆11q lines are profoundly PARPi resistant, SUM149 Ex22 cells are profoundly PARPi sensitive, whereas SUM149 sgGFP cells exhibit intermediate PARPi sensitivity [35].

We measured protein levels of BRCA1-∆11q in this SUM149 cell line panel to assess whether BRCA1-∆11q expression and/or stability were changed when *HUWE1* was silenced. Silencing of *HUWE1* in SUM149-sgGFP control cells resulted in increased steady state levels of BRCA1-∆11q (Fig. 1C), consistent with our hypothesis. To assess whether this could be due to HUWE1-mediated effects on BRCA1-∆11q stability we carried out a cycloheximide chase experiment in SUM149 Ex22 + ∆11q cells, in which new protein synthesis was blocked by cycloheximide. Silencing of HUWE1 increased BRCA1-∆11q protein levels as per our previous observations, and this level remained high two hours after cycloheximide exposure (Fig. 1D). In contrast, control transfected cells showed a lower initial level of BRCA1-∆11q which declined after cycloheximide treatment (Fig. 1D). These results indicated that BRCA1-∆11q protein stability is regulated by HUWE1 in a similar way to full-length BRCA1 [39] and that in the absence of HUWE1, BRCA1-∆11q levels rise. When we exposed SUM149- sgGFP cells (which are able to express BRCA1-∆11q) to PARPi, we found that HUWE1 silencing caused PARPi resistance (Fig. 1E); the resistant phenotype in SUM149-sgGFP cells with HUWE1 silencing was of a similar degree to that seen in SUM149 cells with a BRCA1 reversion (SUM149 Ex11#2C2, Fig. 1E). Conversely, HUWE1 silencing generated only a modest PARPi resistance phenotype in SUM149 Ex22 cells, which because of the exon 22 mutation, are unable to encode the BRCA1-∆11q protein (Fig. 1E). In SUM149 Ex11#2C2, HUWE1 silencing caused a modest increase in PARPi sensitivity at high talazoparib concentrations (Fig. 1E), we assume because HUWE1 also modulates other HR proteins. We also found that inhibiting HUWE1 activity with a small molecule inhibitor [40] (Fig. 1F) or silencing HUWE1 via CRISPR interference in SUM149-TRE-dCas9-KRAB cells, caused PARPi resistance, suggesting that this phenotype is not due to off-target effects of HUWE1 siRNA (Fig. 1G). Protein levels of HUWE1 itself were not affected by PARPi exposure (Supplementary Figure 1A). Taken together, these results suggested that it is a reduction in HUWE1 that enables elevated levels of BRCA1-∆11q and PARP inhibitor resistance in cells that have the ability to encode BRCA1-∆11q but which lack full length BRCA1.

### Silencing of *HUWE1* causes PARPi resistance in cells with *BRCA1* exon 11 mutations

**Fig. 2:**
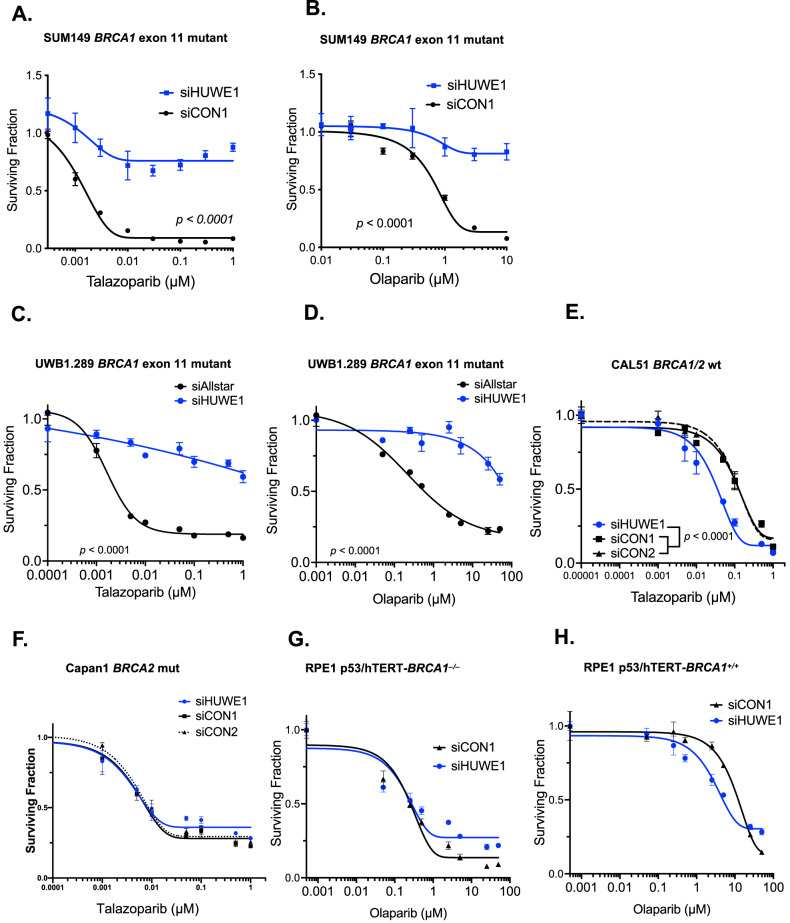
*HUWE1* silencing causes PARPi resistance in *BRCA1* exon 11 mutant cells but not in wild type, *BRCA2*m or *BRCA1* knockout cells. **A**, **B** siHUWE1 causes talazoparib and olaparib resistance in *BRCA1* exon11 mutant SUM149 cells. Cells were transfected with siRNAs and 48 h later, exposed to PARP inhibitors for five days. Cell survival was measured using CellTiter-Glo. **C**, **D** siHUWE1 causes talazoparib and olaparib resistance in *BRCA1* exon11 mutant UWB1.289 cells. **E** HUWE1 silencing does not cause talazoparib resistance in *BRCA1* wild type CAL51 cells. **F** HUWE1 silencing does not cause talazoparib resistance in *BRCA2* mutant Capan1 cells. **G**, **H** Silencing of HUWE1 does not cause pronounced olaparib resistance in *BRCA1* knockout RPE-1 cells (**G**), nor in parental RPE-1 p53/hTERT cells (**H**). Error bars represent standard deviation, *n* ≥ 3.

We confirmed that HUWE1 silencing leads to PARPi resistance using either talazoparib or olaparib in parental SUM149 cells (without sg-GFP, Fig. 2A, B) and in another tumour cell line with a pathogenic exon 11 frameshift *BRCA1* mutation, UWB1.289 (high grade serous ovarian cancer, referred to as UWB1, Fig. 2C, D). When we assessed the generality of the relationship between HUWE1 loss of function and PARPi resistance in additional cell lines, we noted that *BRCA1/2* wild type CAL51 breast tumour cells (Fig. 2E) or *BRCA2* mutant Capan1 pancreatic tumour cells (Fig. 2F) did not display PARPi resistance upon *HUWE1* silencing; in fact, CAL51 cells showed a slight increase in PARPi sensitivity upon HUWE1 silencing (Fig. 2E), suggesting that the effect of HUWE1 on PARPi sensitivity/resistance might be contextualised by the status of BRCA1. However, we also found that RPE-1 retinal pigment epithelial cells with an engineered mutation in *BRCA1* which causes complete loss-of-function and the inability to encode BRCA1-∆11q [41], also did not show a clear PARPi resistance phenotype upon *HUWE1* silencing (Fig. 2G, H). These results supported our hypothesis that the resistance caused by *HUWE1* silencing in *BRCA1* mutant cells only occurs in cells that, by reason of the type of mutation they carry, are able to encode the BRCA1-∆11q splice variant.

To address whether other known mechanisms of PARPi resistance might be operating, we profiled HUWE1 knockdown cells by mass spectrometry. We observed lower levels of HUWE1 and higher levels of known HUWE1 substrates such as MCL1 in knockdown cells. We also noted significant upregulation of EGFR and AURKA (Supplementary Fig. 1B). Expression of other DNA damage response (DDR) components that have been reported to affect PARPi resistance in *BRCA1* mutant cells such as 53PB1, MAD2L2 and PARP1, was not affected by HUWE1 knockdown (Supplementary Fig. 1C, D). To investigate whether the effects on PARP inhibitor resistance might be due to these changes in AURKA or EGFR, we tested whether inhibitors of these proteins (Alisertib and gefitinib respectively) affected talazoparib resistance in *HUWE1* knockdown cells. Although alisertib was synergistic with talazoparib in reverted SUM149 cells, this was independent of HUWE1 status (Supplementary Fig. 1E, H). Neither alisertib nor gefitinib showed synergy with talazoparib in parental (BRCA1 exon 11 mutant) SUM149 cells, regardless of *HUWE1* knockdown (Supplementary Fig. 1F, G, I, J), suggesting that the HUWE1 resistance is not dependent on increased Aurora A or EGFR activity.

### *HUWE1* silencing results in increased RAD51 nuclear foci in BRCA1 exon 11 mutant cells

**Fig. 3:**
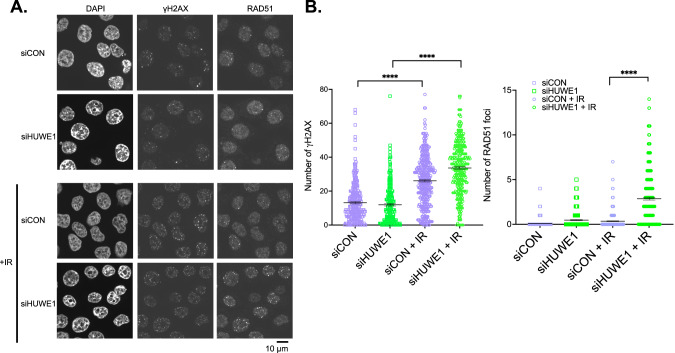
*HUWE1* silencing partially rescues HR capacity in BRCA1-∆11q expressing cells. **A** Confocal microscopy images of IR-induced RAD51 (right) and γH2AX foci (middle panel) from SUM149 cells transfected with HUWE1 siRNA. Cells were transfected with indicated siRNA and irradiated 48 h later. Nuclear foci were detected by immunofluorescence 4 h later and nuclei detected by DAPI staining (left). **B** Quantification of nuclear foci from experiment described in (A). Foci were counted using CellProfiler. *p* values were calculated using two-sided t-test. ****p < 0.0001.

To assess whether the loss of HUWE1 and stabilisation of BRCA1-∆11q led to a reversal of the key DNA repair defect in BRCA1 mutant cells, we assessed the ability of cells to localise the DNA recombinase RAD51 to nuclear foci in response to exogenous DNA damage. SUM149 Parental cells have a reduced ability to form DNA damage induced RAD51 foci, consistent with their hypomorphic BRCA1 mutation, reduced HR capacity and PARPi sensitivity [42] (Fig. 3A, B). However, when we silenced HUWE1, we noted a significant increase in the frequency of cells with DNA damage induced RAD51 foci, suggesting that RAD51 mediated DNA repair was more active (Fig. 3A, B). This data also suggested that clinical assays that estimate HR capacity and subsequent PARPi response by measuring nuclear RAD51 [43] would be likely to detect resistance caused by loss of HUWE1 and subsequent upregulation of BRCA1-∆11q.

### *HUWE1* mutations identified in a case of *BRCA1m* high grade serous ovarian cancer with acquired resistance to olaparib

**Fig. 4:**
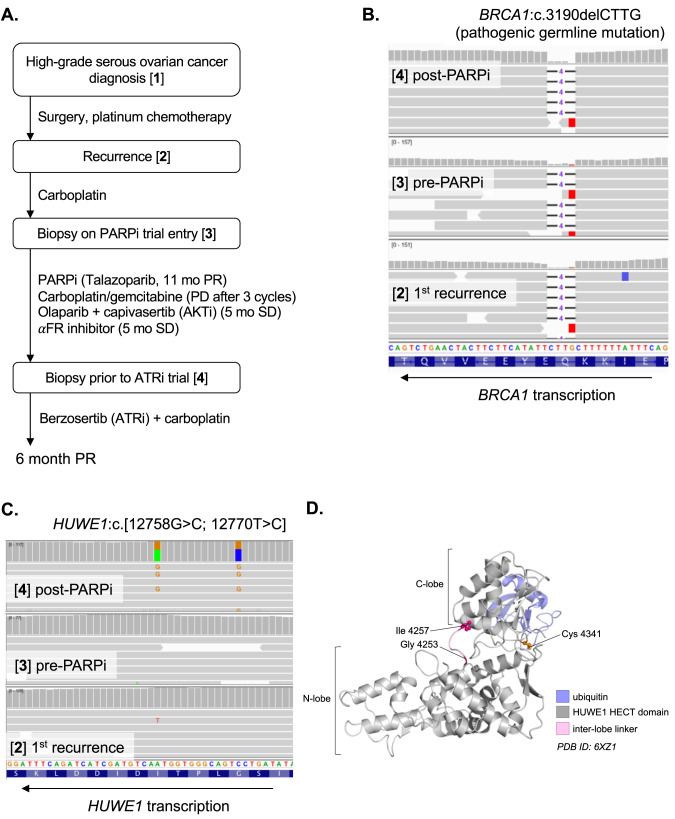
Development of PARPi-refractory disease in a patient with *HUWE1* mutations. **A** Schematic of patient treatment journey and biopsies analysed. **B** No evidence of *BRCA1* reversion across all the patient biopsies. No secondary mutations were called using standard somatic variant calling or by manual inspection in IGV (Integrated Genomics Viewer; view around pathogenic mutation shown). **C** Two missense *HUWE1* mutations in *cis* identified only in the post-PARPi tumour biopsy (G4253A;I4257T). Alignments for reads at the mutation site in HUWE1 are shown. **D** Mutations affect catalytic domain but not catalytic site directly. Structure of the HUWE1 catalytic HECT domain [57] (PDB:6XZ1) showing residues affected by mutations (pink) in relation to the catalytic cysteine reside (orange).

In seeking to understand whether HUWE1-mediated PARPi resistance occurs in the clinical disease as well as in cell lines, we analysed DNA sequencing from tumor and ctDNA from a 54 year old patient with high grade serous ovarian cancer who received the PARP inhibitor olaparib as part of the ComPAKT Phase Ib trial (in combination with the AKT inhibitor capivasertib [44]). The patient initially presented with a *BRCA1* mutant (*BRCA1*:c.3190delCTTG) high grade serous ovarian cancer and had initial debulking surgery before receiving standard of care platinum-based chemotherapy. Her initial anti-tumour response lasted 16 months until disease recurred. Three years after her initial diagnosis she was enrolled on a PARP inhibitor (talazoparib) trial and achieved a RECIST partial response lasting 11 months, after which her disease progressed. The patient then received a further line of carboplatin/gemcitabine combination chemotherapy, with disease progression noted after three cycles of treatment, making her platinum-refractory. The patient then entered the ComPAKT phase Ib trial of olaparib and the AKT inhibitor capivasertib [44] and achieved RECIST stable disease with minor tumor regression to treatment lasting five months, before discontinuing trial for disease progression. The patient subsequently received an α-folate receptor inhibitor for five further months, after which she entered a phase I trial of the ATR inhibitor berzosertib in combination with carboplatin [45] (Fig. 4).

Several biopsies were taken during the course of treatment, including one prior to the first PARP inhibitor trial and another prior to entry into the ATR inhibitor study (i.e. after progression on two PARP inhibitor-containing regimens and platinum-based chemotherapy; Fig. 4A). Whole exome sequencing was performed on DNA extracted from FFPE tissue from this series of biopsies. In the first instance, we assessed whether reversion mutations in *BRCA1*, the most clinically validated mechanism of PARPi and platinum salt resistance in *BRCA1* mutant cancers [10], could explain the PARPi resistance in this individual. In this case, no additional *BRCA1* mutations were identified in any of the samples analysed, and the variant allele frequency (VAF) of the original germline *BRCA1* 4-bp deletion was close to 1 in all tumour samples, consistent with loss-of-heterozygosity (LOH) and maintenance of *BRCA1* loss throughout the progression of disease (Fig. 4B). The allele frequency of a deleterious *TP53* mutation (p.Y220C) also suggested high tumour content (range 0.78-0.95 across all biopsies). Manual inspection of DNA sequence alignments did not yield any evidence of secondary mutations that might restore the reading frame of *BRCA1*, nor did any ctDNA analysis carried out as part of the CompAKT trial [44]. Therefore, direct restoration of *BRCA1* at the genetic level via reversion was unlikely to be the cause of PARPi resistance in this patient.

Interestingly, we found two closely linked somatic missense mutations in *HUWE1* (c.[12758 G > C; 12770 T > C], p.[4253 G > A; 4257I > T]; Fig. 4C) that were unique to the biopsy taken after progression on olaparib. These mutations were in the catalytic HECT domain of HUWE1, although not in residues directly involved in catalysis (Fig. 4D). Very few truncating mutations have been reported in *HUWE1* [46, 47] which, along with the embryonic lethality of *Huwe1* knockout mice [48], suggests that HUWE1 is an essential gene in the whole organism context. However, it is possible that mutations in HUWE1 such as p.4253 G > A and p.4257I > T cause partial loss of HUWE1 function.

## Discussion

The data presented above indicate that loss of HUWE1 function provides one route to an increase in steady-state levels of the BRCA1 splice variant BRCA1-∆11q and PARPi resistance. Notably, this form of HUWE1-mediated resistance appeared to be particular to tumour cells with BRCA1 exon 11 truncating mutations that are able to express the BRCA1-∆11q protein and was not seen, for example, in tumour cells with *BRCA1* exon 22 mutations or in *BRCA2* mutant cells. This suggests that this might be one example of where the mechanism of drug resistance might be highly contextualised not just by the identity of the cancer driver gene that originally causes drug sensitivity, but also by the particular mutation type present in the driver gene.

In other contexts, HUWE1 has previously been associated with PARPi resistance. For example, in a forward genetic screen for PARP inhibitor resistance in an engineered HeLa cell line with a *BRCA2* deficiency, *HUWE1* suppression increased PARP inhibitor resistance [49]. *HUWE1*-silenced BRCA2 mutant cells had an increased level of RAD51 mRNA and protein, although the precise mechanism of resistance in this situation is not clear, nor whether this might be in any way related to the observations here, where we find the effect is specific to BRCA1-∆11q. The most likely explanation for this specificity is that the modification site that regulates BRCA1 ubiquitylation and degradation mediated by HUWE1 is still present in the ∆11q variant, allowing it to also be regulated by HUWE1. This is consistent with previous data suggesting that the modification occurs in the region of BRCA1 between amino acids 1-167, which is present in both full length BRCA1 and ∆11q [39]. There is evidence for reacquisition of BRCA1 function in tumours that become resistant to PARP inhibitors or platinum, and although BRCA1-∆11q has been shown to drive resistance in overexpression experiments (such as the SUM149 Ex22 + ∆11q cell line used here [35]), it is unclear how tumours would achieve such levels in practice. The evidence we present here suggests that HUWE1 limits BRCA1-∆11q levels and loss of HUWE1 is sufficient for clinically meaningful resistance – i.e. similar to that caused by a *BRCA1* reversion mutation (Fig. 1E).

*HUWE1* is not a commonly mutated cancer gene; indeed *HUWE1* mutations are rare in the population as a whole and CRISPR-Cas9 mutagenesis of *HUWE1* in > 900 tumour cell lines (in the DepMap dataset [50]) suggest that complete loss-of-function is lethal. However, our observation of *HUWE1* mutations in the context of acquired resistance to PARP inhibitors suggests that more subtle *HUWE1* mutations can occur in patients. Taken together with the effects we have described on PARPi resistance in cell lines, our findings support the further investigation of *HUWE1* mutations in PARP inhibitor resistant patients with *BRCA1* exon 11 mutations.

## Materials and Methods

### Patient biopsies

Tumor biopsies were obtained and analyzed as part of previously conducted clinical trials as detailed in the main text [44, 45].

### DNA sequencing analysis

DNA extracted from FFPE tumours and normal tissue was sequenced using an exome capture panel. BWA (version 0.7.5a) was used to align reads to the human reference genome (GRCh37). PCR duplicates were removed prior to further processing and variant detection. Variant calling was performed using MuTect (version 1.1.4) and the Genome Analysis Tool Kit (GATK) (version 2.7-2) Broad Best Practices Pipeline with standard settings. Variants were excluded with genotype qualities below 20 and those covered by fewer than ten reads in either sample. Mutations and small indels were selected from the complete set of variants called using the GATK unified genotyper based on differences in the variant allele fractions observed in the tumour and germline/normal exome sequence data.

### Cell culture, siRNA and dose-response survival assays

SUM149 cell lines were maintained in Ham’s F12 with 5% fetal calf serum (FCS), 1 µg/ml hydrocortisone and 5 µg/ml insulin. SUM149 and the derivatives used here have been previously described [14, 33, 42, 51]. COV362, CAL51 and RPE1 cell lines were maintained in DMEM + 10% FCS [51]. UWB1.289 cells [52] were maintained in 50:50 RPMI:MEGM with 3% FCS. Capan1 cells were maintained in IMDM + 20% FCS. Cells were transfected with siRNA using Lipofectamine 3000 in 384-well plates (250–500 cells per well) and exposed to drugs 48 h later. After five further days, cell growth was assessed using CellTiter-Glo. Olaparib, talazoparib, alisertib and gefitinib were obtained from SelleckChem and stock solutions made in DMSO. Dose-response assays were carried out in 384-well plates using an Echo acoustic dispenser from stock plates. The final DMSO percentage ( < 0.1% v/v) was equalised across all drug concentrations.

siRNA used were: siHUWE1 (Dharmacon M-007185-01-0005), siBRCA1 (Dharmacon M-003461-02-0005), siCON (Dharmacon D-001210-01-05), Allstar (Qiagen SI03650318).

### CRISPR reagents

Constructs expressing CRISPR interference guides targeting *HUWE1* were made by cloning double stranded oligonucleotides into the *Bbs*I target site of pKLV2-U6gRNA5(BbsI)-PGKpuro2AmBFP-W as previously described [53]. Target sites used: sgRNA2, GGTCCGGTAGAGGTTCTCGC; sgRNA3 GACTGCGGCGGCGACAACGG.

### Western blot

The following antibodies were used: HUWE1, A300-486A, Bethyl Laboratories (1:1000 dilution); Lamin B, ab16048, abcam (1:1000); Tubulin, ab7291, abcam (1:5000); Vinculin, sc-73614 (7F9), Santa Cruz (1:500); BRCA1, OP92 (MS110; 1:500), Sigma; Cas9, 7A9-3A3, Novus Biologicals (1:1000). Western blots were scanned using a BioRad ChemiDoc or LiCor Odyssey. Uncropped scans of all blots are shown in the Supplementary Data. For detection of BRCA1, nuclear extracts were prepared using the Subcellular Protein Fractionation Kit (Thermo Scientific #78840).

### Immunofluorescence

Cells were treated as indicated. Cells were transfected with siRNA as above in 6-well plates. The next day, cells were seeded in black 96-well plates at 20,000 cells per well for irradiation the following day (48 h post transfection). For ionising radiation, cells were exposed to 10 Gy using an X-ray source and allowed to recover at 37 C for 4 h prior to fixation and imaging. Cells were prepared and stained as previously described [54], and imaged using a spinning disk confocal microscope. Foci were counted automatically using CellProfiler software. Antibodies used were: RAD51, ab133534, abcam (1:1000); γH2AX, 05-636, Millipore (1:1000).

### Protein stability experiments

Cells were transfected with siRNA as above and exposed to cycloheximide (150 µg/ml) 48 h later. Whole cell lysates from samples taken at subsequent timepoints were used in Western blots to analyse protein levels.

### Mass spectrometry

Protein lysates were prepared for mass spectrometry and analysed as previously described [55].

## Supplementary information

Supplementary Figures

## Data Availability

All data are contained in the manuscript or available from the corresponding authors on reasonable request. The mass spectrometry proteomics data have been deposited to the ProteomeXchange Consortium via the PRIDE [56] partner repository with the dataset identifier PXD040430.

## References

[1] Lord CJ, Ashworth A (2017). PARP inhibitors: Synthetic lethality in the clinic. Science.

[2] Robson M, Im S-A, Senkus E, Xu B, Domchek SM, Masuda N (2017). Olaparib for Metastatic Breast Cancer in Patients with a Germline BRCAMutation. N. Engl J Med.

[3] Tutt ANJ, Garber JE, Kaufman B, Viale G, Fumagalli D, Rastogi P, et al. Adjuvant Olaparib for Patients with BRCA1- or BRCA2-Mutated Breast Cancer. N Engl J Med. 2021. 10.1056/NEJMoa2105215. 10.1056/NEJMoa2105215 PMC9126186 34081848

[4] Litton JK, Rugo HS, Ettl J, Hurvitz SA, Gonçalves A, Lee K-H (2018). Talazoparib in Patients with Advanced Breast Cancer and a Germline BRCA Mutation. N Engl J Med.

[5] Mirza MR, Monk BJ, Herrstedt J, Oza AM, Mahner S, Redondo A (2016). Niraparib Maintenance Therapy in Platinum-Sensitive, Recurrent Ovarian Cancer. N Engl J Med.

[6] Coleman RL, Oza AM, Lorusso D, Aghajanian C, Oaknin A, Dean A (2017). Rucaparib maintenance treatment for recurrent ovarian carcinoma after response to platinum therapy (ARIEL3): a randomised, double-blind, placebo-controlled, phase 3 trial. Lancet.

[7] Pujade-Lauraine E, Ledermann JA, Selle F, Gebski V, Penson RT, Oza AM (2017). Olaparib tablets as maintenance therapy in patients with platinum-sensitive, relapsed ovarian cancer and a BRCA1/2 mutation (SOLO2/ENGOT-Ov21): a double-blind, randomised, placebo-controlled, phase 3 trial. Lancet Oncol.

[8] Ledermann J, Harter P, Gourley C, Friedlander M, Vergote I, Rustin G (2012). Olaparib maintenance therapy in platinum-sensitive relapsed ovarian cancer. N. Engl J Med.

[9] Swisher EM, Kwan TT, Oza AM, Tinker AV, Ray-Coquard I, Oaknin A (2021). Molecular and clinical determinants of response and resistance to rucaparib for recurrent ovarian cancer treatment in ARIEL2 (Parts 1 and 2). Nat Commun.

[10] Pettitt SJ, Frankum JR, Punta M, Lise S, Alexander J, Chen Y (2020). Clinical BRCA1/2 Reversion Analysis Identifies Hotspot Mutations and Predicted Neoantigens Associated with Therapy Resistance. Cancer Discov.

[11] Edwards SL, Brough R, Lord CJ, Natrajan R, Vatcheva R, Levine DA (2008). Resistance to therapy caused by intragenic deletion in BRCA2. Nature.

[12] Tobalina L, Armenia J, Irving E, O’Connor MJ, Forment JV (2021). A meta-analysis of reversion mutations in BRCA genes identifies signatures of DNA end-joining repair mechanisms driving therapy resistance. Ann Oncol.

[13] Domchek SM (2017). Reversion Mutations with Clinical Use of PARP Inhibitors: Many Genes, Many Versions. Cancer Discov.

[14] Pettitt SJ, Krastev DB, Brandsma I, Dréan A, Song F, Aleksandrov R (2018). Genome-wide and high-density CRISPR-Cas9 screens identify point mutations in PARP1 causing PARP inhibitor resistance. Nat Commun.

[15] Pettitt SJ, Rehman FL, Bajrami I, Brough R, Wallberg F, Kozarewa I (2013). A Genetic Screen Using the PiggyBac Transposon in Haploid Cells Identifies Parp1 as a Mediator of Olaparib Toxicity. PLoS One.

[16] Gogola E, Duarte AA, de Ruiter JR, Wiegant WW, Schmid JA, de Bruijn R (2018). Selective Loss of PARG Restores PARylation and Counteracts PARP Inhibitor-Mediated Synthetic Lethality. Cancer Cell.

[17] Jaspers JE, Kersbergen A, Boon U, Sol W, van Deemter L, Zander SA (2013). Loss of 53BP1 causes PARP inhibitor resistance in Brca1-mutated mouse mammary tumors. Cancer Discov.

[18] Xu G, Chapman JR, Brandsma I, Yuan J, Mistrik M, Bouwman P (2015). REV7 counteracts DNA double-strand break resection and affects PARP inhibition. Nature.

[19] Noordermeer SM, Adam S, Setiaputra D, Barazas M, Pettitt SJ, Ling AK (2018). The shieldin complex mediates 53BP1-dependent DNA repair. Nature.

[20] Gupta R, Somyajit K, Narita T, Maskey E, Stanlie A, Kremer M (2018). DNA Repair Network Analysis Reveals Shieldin as a Key Regulator of NHEJ and PARP Inhibitor Sensitivity. Cell.

[21] Ghezraoui H, Oliveira C, Becker JR, Bilham K, Moralli D, Anzilotti C (2018). 53BP1 cooperation with the REV7-shieldin complex underpins DNA structure-specific NHEJ. Nature.

[22] Dev H, Chiang T-WW, Lescale C, de Krijger I, Martin AG, Pilger D (2018). Shieldin complex promotes DNA end-joining and counters homologous recombination in BRCA1-null cells. Nat Cell Biol.

[23] Mirman Z, Lottersberger F, Takai H, Kibe T, Gong Y, Takai K (2018). 53BP1-RIF1-shieldin counteracts DSB resection through CST- and Polα-dependent fill-in. Nature.

[24] Barazas M, Annunziato S, Pettitt SJ, de Krijger I, Ghezraoui H, Roobol SJ, et al. The CST Complex Mediates End Protection at Double-Strand Breaks and Promotes PARP Inhibitor Sensitivity in BRCA1-Deficient Cells. CellReports. 2018;23:2107–18. 10.1016/j.celrep.2018.04.046 PMC5972230 29768208

[25] Rottenberg S, Jaspers JE, Kersbergen A, van der Burg E, Nygren AOH, Zander SAL (2008). High sensitivity of BRCA1-deficient mammary tumors to the PARP inhibitor AZD2281 alone and in combination with platinum drugs. Proc Natl Acad Sci.

[26] Christie EL, Pattnaik S, Beach J, Copeland A, Rashoo N, Fereday S (2019). Multiple ABCB1 transcriptional fusions in drug resistant high-grade serous ovarian and breast cancer. Nat Commun.

[27] Kondrashova O, Nguyen M, Shield-Artin K, Tinker AV, Teng NNH, Harrell MI (2017). Secondary Somatic Mutations Restoring RAD51C and RAD51D Associated with Acquired Resistance to the PARP Inhibitor Rucaparib in High-Grade Ovarian Carcinoma. Cancer Discov.

[28] Patel JN, Braicu I, Timms KM, Solimeno C, Tshiaba P, Reid J (2018). Characterisation of homologous recombination deficiency in paired primary and recurrent high-grade serous ovarian cancer. Br J Cancer.

[29] Simmons AD, Nguyen M, Pintus E (2020). Polyclonal BRCA2 mutations following carboplatin treatment confer resistance to the PARP inhibitor rucaparib in a patient with mCRPC: a case report. BMC Cancer.

[30] Waks AG, Cohen O, Kochupurakkal B, Kim D, Dunn CE, Buendia Buendia J (2020). Reversion and non-reversion mechanisms of resistance to PARP inhibitor or platinum chemotherapy in BRCA1/2-mutant metastatic breast cancer. Ann Oncol.

[31] Buisson M, Anczuków O, Zetoune AB, Ware MD, Mazoyer S (2006). The 185delAG mutation (c.68_69delAG) in the BRCA1 gene triggers translation reinitiation at a downstream AUG codon. Hum Mutat.

[32] Drost R, Dhillon KK, van der Gulden H, van der Heijden I, Brandsma I, Cruz C (2016). BRCA1185delAG tumors may acquire therapy resistance through expression of RING-less BRCA1. J Clin Invest.

[33] Wang Y, Krais JJ, Bernhardy AJ, Nicolas E, Cai KQ, Harrell MI (2016). RING domain-deficient BRCA1 promotes PARP inhibitor and platinum resistance. J Clin Invest.

[34] Johnson N, Johnson SF, Yao W, Li Y-C, Choi Y-E, Bernhardy AJ (2013). Stabilization of mutant BRCA1 protein confers PARP inhibitor and platinum resistance. Proc Natl Acad Sci USA.

[35] Wang Y, Bernhardy AJ, Cruz C, Krais JJ, Nacson J, Nicolas E (2016). The BRCA1- 11q Alternative Splice Isoform Bypasses Germline Mutations and Promotes Therapeutic Resistance to PARP Inhibition and Cisplatin. Cancer Res.

[36] Tammaro C, Raponi M, Wilson DI, Baralle D (2012). BRCA1 exon 11 alternative splicing, multiple functions and the association with cancer. Biochem Soc Trans.

[37] Wenmaekers S, Viergever BJ, Kumar G, Kranenburg O, Black PC, Daugaard M, et al. A Potential Role for HUWE1 in Modulating Cisplatin Sensitivity. Cells 2021;10. 10.3390/cells10051262. 10.3390/cells10051262 PMC8160634 34065298

[38] Choe KN, Nicolae CM, Constantin D, Imamura Kawasawa Y, Delgado-Diaz MR, De S (2016). HUWE 1 interacts with PCNA to alleviate replication stress. EMBO Rep.

[39] Wang X, Lu G, Li L, Yi J, Yan K, Wang Y (2014). HUWE1 interacts with BRCA1 and promotes its degradation in the ubiquitin-proteasome pathway. Biochem Biophys Res Commun.

[40] Peter S, Bultinck J, Myant K, Jaenicke LA, Walz S, Müller J (2014). Tumor cell-specific inhibition of MYC function using small molecule inhibitors of the HUWE1 ubiquitin ligase. EMBO Mol Med.

[41] Zimmermann M, Murina O, Reijns MAM, Agathanggelou A, Challis R (2018). CRISPR screens identify genomic ribonucleotides as a source of PARP-trapping lesions. Nature.

[42] Dréan A, Williamson CT, Brough R, Brandsma I, Menon M, Konde A (2017). Modeling Therapy Resistance in BRCA1/2-Mutant Cancers. Mol Cancer Ther.

[43] Cruz C, Castroviejo-Bermejo M, Gutiérrez-Enríquez S, Llop-Guevara A, Ibrahim YH, Gris-Oliver A (2018). RAD51 foci as a functional biomarker of homologous recombination repair and PARP inhibitor resistance in germline BRCA-mutated breast cancer. Ann Oncol.

[44] Yap TA, Kristeleit R, Michalarea V, Pettitt SJ, Lim JSJ, Carreira S, et al. Phase I trial of the poly(ADP-ribose) polymerase (PARP) inhibitor olaparib and AKT inhibitor capivasertib in patients with BRCA1/2 and non-BRCA1/2 mutant cancers. Cancer Discov. 2020. 10.1158/2159-8290.CD-20-0163. 10.1158/2159-8290.CD-20-0163 PMC7611385 32532747

[45] Yap TA, O’Carrigan B, Penney MS, Lim JS, Brown JS, de Miguel Luken MJ (2020). Phase I Trial of First-in-Class ATR Inhibitor M6620 (VX-970) as Monotherapy or in Combination With Carboplatin in Patients With Advanced Solid Tumors. J Clin Oncol.

[46] Moortgat S, Berland S, Aukrust I, Maystadt I, Baker L, Benoit V (2018). HUWE1 variants cause dominant X-linked intellectual disability: a clinical study of 21 patients. Eur J Hum Genet.

[47] Lek M, Karczewski KJ, Minikel EV, Samocha KE, Banks E, Fennell T (2016). Analysis of protein-coding genetic variation in 60,706 humans. Nature.

[48] Hao Z, Duncan GS, Su Y-W, Li WY, Silvester J, Hong C (2012). The E3 ubiquitin ligase Mule acts through the ATM-p53 axis to maintain B lymphocyte homeostasis. J Exp Med.

[49] Clements KE, Schleicher EM, Thakar T, Hale A, Dhoonmoon A, Tolman NJ (2020). Identification of regulators of poly-ADP-ribose polymerase inhibitor response through complementary CRISPR knockout and activation screens. Nat Commun.

[50] Tsherniak A, Vazquez F, Montgomery PG, Weir BA, Kryukov G, Cowley GS (2017). Defining a Cancer Dependency Map. Cell.

[51] Elstrodt F, Hollestelle A, Nagel JHA, Gorin M, Wasielewski M, van den Ouweland A (2006). BRCA1 mutation analysis of 41 human breast cancer cell lines reveals three new deleterious mutants. Cancer Res.

[52] DelloRusso C, Welcsh PL, Wang W, Garcia RL, King M-C, Swisher EM (2007). Functional characterization of a novel BRCA1-null ovarian cancer cell line in response to ionizing radiation. Mol Cancer Res.

[53] Tzelepis K, Koike-Yusa H, De Braekeleer E, Li Y, Metzakopian E, Dovey OM (2016). A CRISPR Dropout Screen Identifies Genetic Vulnerabilities and Therapeutic Targets in Acute Myeloid. Leuk Cell Rep.

[54] Zatreanu D, Robinson HMR, Alkhatib O, Boursier M, Finch H, Geo L (2021). Polθ inhibitors elicit BRCA-gene synthetic lethality and target PARP inhibitor resistance. Nat Commun.

[55] Chabanon RM, Morel D, Eychenne T, Colmet-Daage L, Bajrami I, Dorvault N (2021). PBRM1 Deficiency Confers Synthetic Lethality to DNA Repair Inhibitors in Cancer. Cancer Res.

[56] Perez-Riverol Y, Bai J, Bandla C, García-Seisdedos D, Hewapathirana S, Kamatchinathan S (2022). The PRIDE database resources in 2022: a hub for mass spectrometry-based proteomics evidences. Nucleic Acids Res.

[57] Sander B, Xu W, Eilers M, Popov N, Lorenz S. A conformational switch regulates the ubiquitin ligase HUWE1. Elife 2017;6. 10.7554/eLife.21036. 10.7554/eLife.21036 PMC5308896 28193319

