# Supplementary material for: A HUWE1 defect causes PARP inhibitor resistance by modulating the BRCA1-∆11q splice variant
Source: Oncogene. 2023 Jul 25;42(36):2701–9. doi: 10.1038/s41388-023-02782-8 (PMC10473960; doi:10.1038/s41388-023-02782-8)
Supplement: Supplementary file 1 — Supplementary Figures [file 41388_2023_2782_MOESM1_ESM.pdf]

Supplementary Figure 1. Resistance is not caused by changes in other HUWE1 substrates (legend on following page)

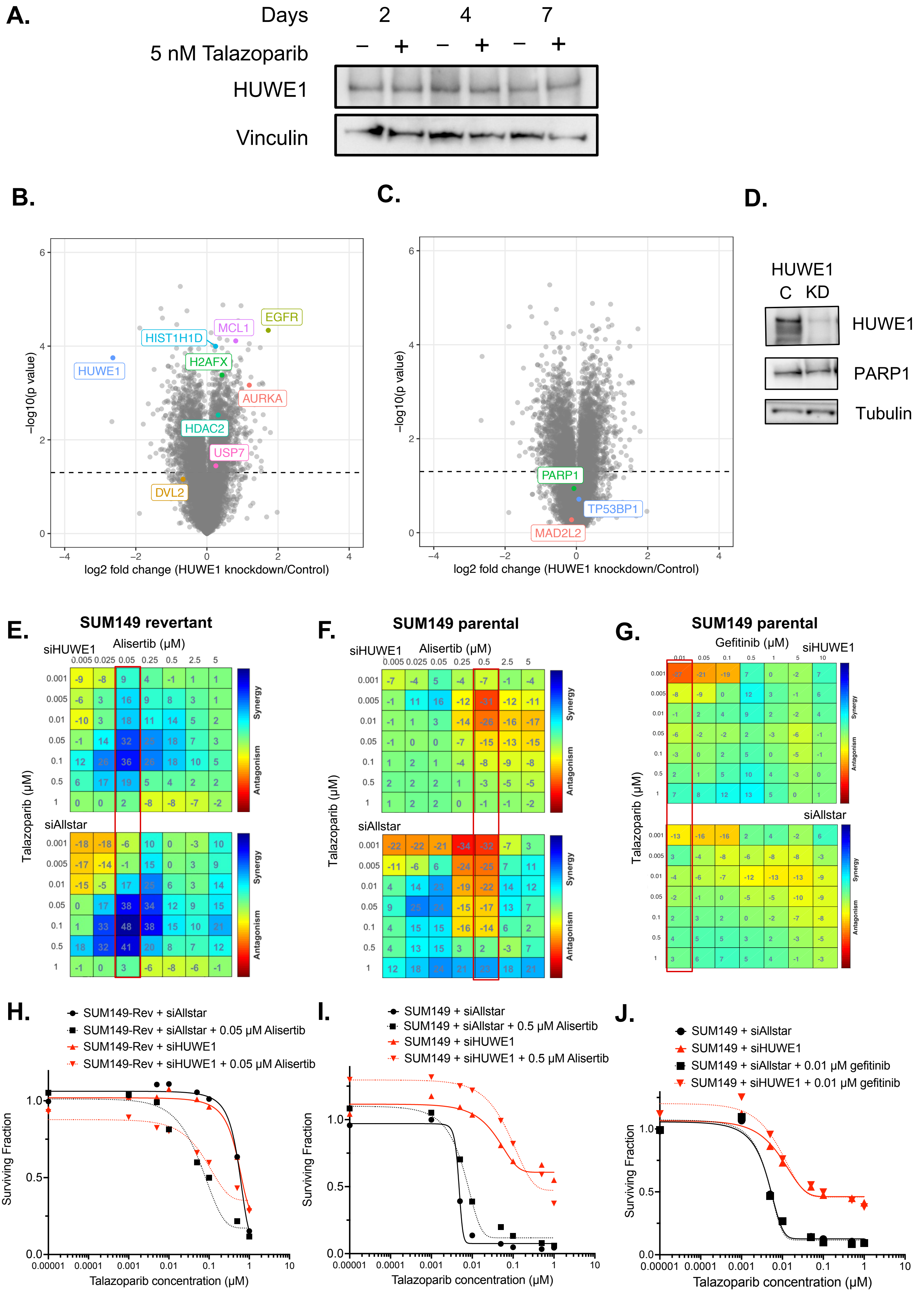

Supplementary Figure 1 | Resistance is not explained by changes in other HUWE1 substrates. **A.** HUWE1 expression levels do not change upon exposure to PARP inhibitor. SUM149 cells were exposed to talazoparib for the indicated times and HUWE1 levels assessed by Western blot. **B.** Volcano plot illustrating differential abundance of proteins in SUM149 cells with HUWE1 knockdown compared to control. Aurora A, EGFR, HUWE1 and selected known HUWE1 targets are highlighted. **C.** Volcano plot as in B highlighting known determinants of PARPi resistance that were detectable in the experiment. **D.** No change in PARP1 expression after HUWE1 KD in SUM149 confirmed by Western Blot. C, siCONTROL; KD, siHUWE1. **E-F** Synergy grids for different doses of talazoparib combined with alisertib in the indicated cell lines. SUM149 parental (*BRCA1* exon 11 mutation) or revertant cell lines were transfected with siRNAs and plated for dose-response survival analysis using a range of doses of talazoparib and alisertib. Surviving fraction was assessed using CellTiter Glo. Bliss synergy scores, which measure whether the survival with the combination of drugs differs from that expected from the additive effects of each individual concentration, are plotted as a heatmap. **G** Synergy grid for talazoparib in combination with gefitinib in SUM149 cells. **H, I.** Dose response curves extracted from the concentration of alisertib indicated by the red box in E, F. Aurora A inhibition is synergistic with talazoparib but is not specific to HUWE1 depletion. **J.** Dose response curve from panel G for 0.01 μM gefitinib No effect of EGFR inhibition on talazoparib sensitivity regardless of HUWE1 knockdown.

Supplementary Figure 2. Uncropped Western Blots

A.

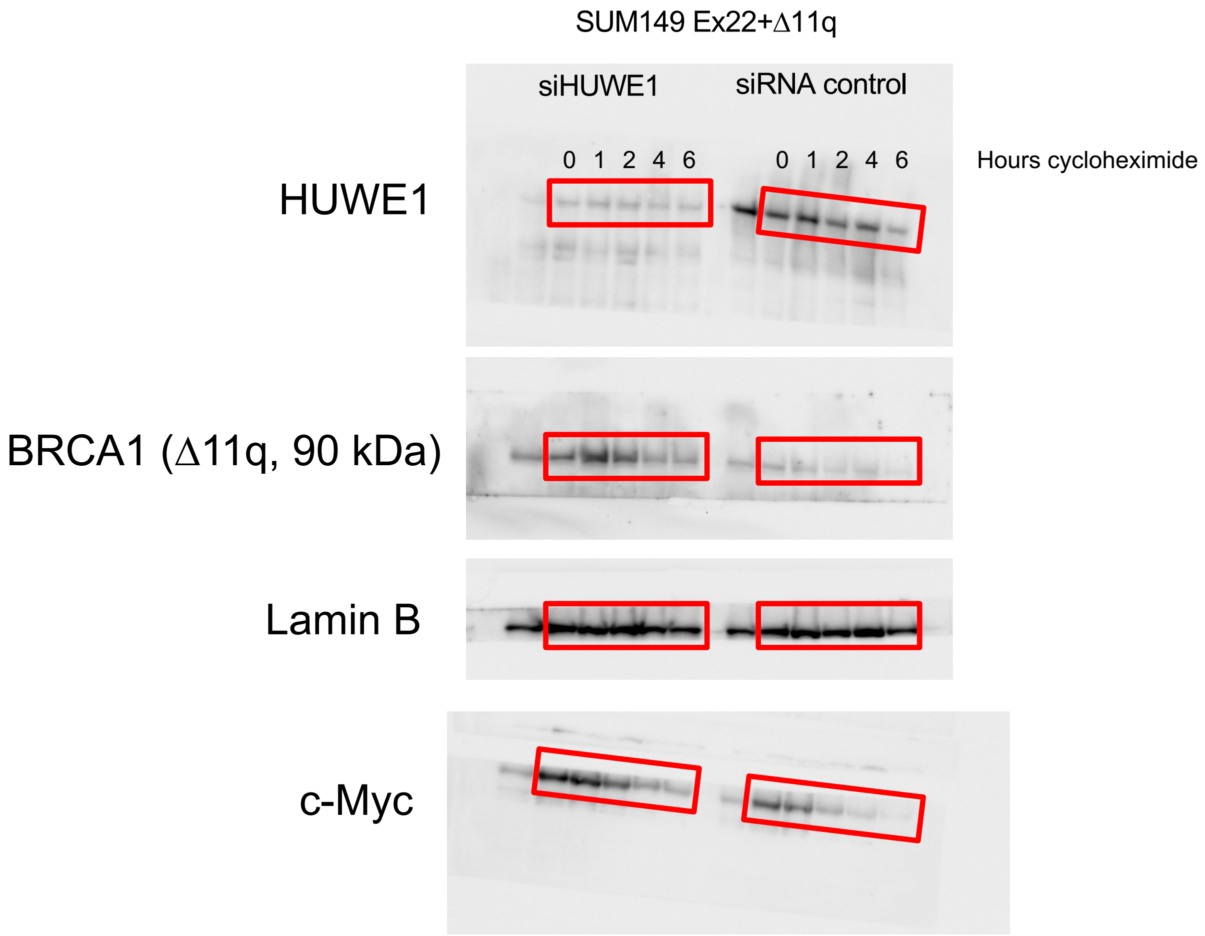

Supplementary Figure 2 | Uncropped Western Blots for Figure 1D. Boxes indicate regions shown on main figure.
